# Data on acoustic behaviour of coconut fibre-reinforced concrete

**DOI:** 10.1016/j.dib.2018.10.133

**Published:** 2018-10-31

**Authors:** Bamigboye Gideon Olukunle, Ngene Ben Uche, Apata Odera Efomo, Adeyemi Gideon, Jolayemi Kayode Joshua

**Affiliations:** Department of Civil Engineering, Covenant University, Ota, Ogun State, Nigeria

## Abstract

The data presented in this research show the results of the experimental study of acoustic behaviour of coconut fibre-reinforced concrete (CFRC). The CFRC was added in percentages of 0.25%, 0.5%, 0.75% and 1% of cement. The acoustic test was conducted to determine the amount of sound that can be absorbed by the fibre. The data showed that the concrete reinforced with coconut fibre had the highest sound absorptive power, of which fibre treated absorbed more sound than other fibres, this is because washing of fibre increase the lignin content which is responsible for the sound absorbing property. The data also showed that the curing time had no effect on the absorbing property of the CFRC. The data presented will be useful in the construction of sound proof reinforced concrete slabs, walls and other elements.

**Specifications table**

**Table:** 

Subject area	*Coconut fibre, acoustic, reinforced concrete, compressive strength, cement, concrete*
More specific subject area	*Concrete, acoustic, coconut fibre, compressive strength.*
Type of data	*Figures and graphs*
How data were acquired	*Laboratory experiment and use of relevant standards*
Data format	*Raw and analyzed*
Experimental factors	*The extraction process of fibre was done by beating the back of the husk in the dry state and by soaking in water and chemical. The percentage of concentration of NaOH used was 1%. The former was done to ensure that fibre remained in the untreated state for use in concrete.*
Experimental features	*The acoustic data of coconut fibre-reinforced concrete were determined by subjecting a concrete panel (15 cm* × *15 cm* ×* 15 cm) produced from 1:3:3 mix ratio to a wooden box together with loudspeaker to amplify generated frequency. Digital sound level meter was used to measure the sound frequency.*
Data source location	*Lagos and Ogun States, Nigeria.*
Data accessibility	*The data are available within this article.*

**Value of the data**

• These data will be useful to researchers working on incorporating coconut fibre on concrete.
• The data presented show that incorporating fibre in concrete improved the slump because the fibre absorbed some of the water during mixing.
• The data are valuable because they show that coconut fibre absorbed some amount of sound. Also, distance of propagation should be considered in further studies.
• The data obtained in this study solve the issue of noise affecting the occupants of buildings located in urban and rural areas.
• The data presented in this study provide an affordable and functional replacement for sound proof materials as opposed to importing of sound absorbing materials.

## 1 Data

**Fig. 1:**
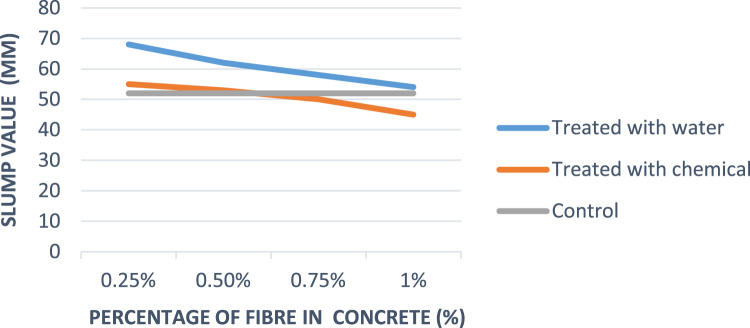
Graph showing slump value.

**Fig. 2:**
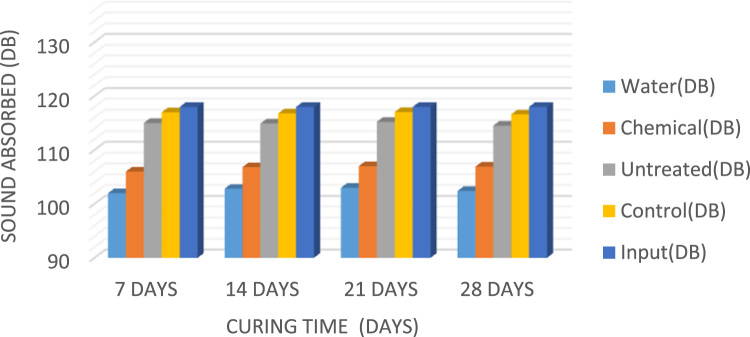
Bar chart showing the sound absorbing value of 0.25% CFRC.

**Fig. 3:**
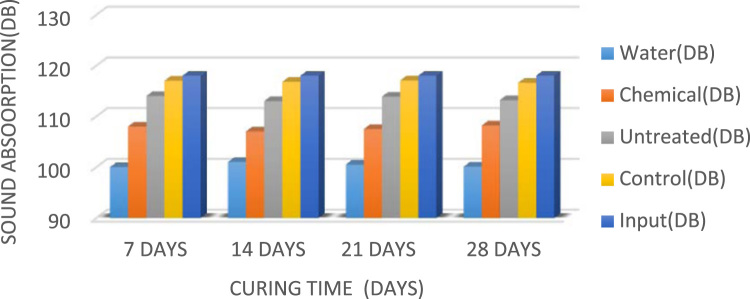
Bar chart showing the sound absorbing value of 0.5% CFRC.

**Fig. 4:**
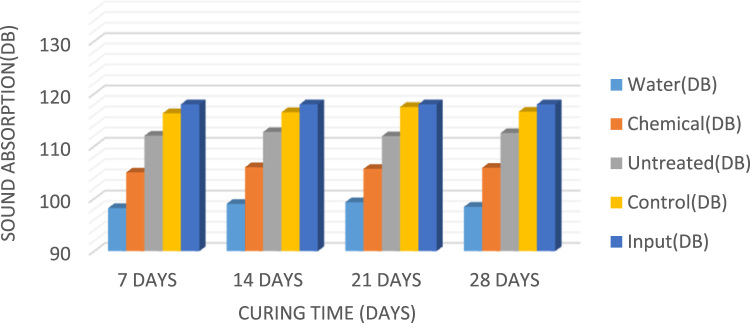
Bar chart showing the sound absorbing value of 0.75% CFRC.

Composite material is defined as a unique combination of the fibre and the concrete matrix where function of the fibre is to withstand load and make the composite stiffer meanwhile matrix is a binder which holds the fibre in place [1]. A fibre-free concrete would develop cracks; however, the addition of fibre would improve and reduce the development of cracks [2]. The natural fibres have been gaining attention in the construction industry compared to the synthetic fibres because of their renewable nature, high availability at low cost, high stiffness, and the encouraging use in different applications [3], [4], [5]. Abbass [6] reported that coconut fibres have excellent characteristics that could be used in construction industry for drainage, filtration, and reinforcement. Currently, most buildings make use of synthetic fibre such as glass and mineral fibre as sound proof materials; the growing health risk of these synthetic fibre has led to the search for a material which is of little or no risk to human health [7]. Xiaoning and Xiong [8] in the study of acoustic absorption properties of fibrous materials concluded that airflow resistivity was an important factor when considering the acoustic behavior of fibrous assemblies. However, in this data presentation, the acoustic behavior would be taken into consideration in converting waste to wealth and in determining the acoustic behaviour of the coconut fibre as a construction material. Fig. 1 present the slum values of fibre treated with water, chemical and that of control while Fig. 2, Fig. 3, Fig. 4 present the sound absorbing value of 0.25–0.75% CFRC.

## 2 Experimental design, materials, and methods

Portland cement of the Dangote brand in conformity with [9] standard was used to generate these data. A combination of ½ in. and ¾ in. coarse aggregate were used while river sand was used as fine aggregates in line with [10], [11]. Batching of concrete was by weight with 1:3:3 mix ratio. The fibre added was calculated using a percentage of the weight of cement. Tap water gotten from the municipal water mains of Covenant University was used for the study. The fibre used for this experiment was the coconut fibre and it was gotten from Seme [6°, 22′, 32.99″N] and [2°, 43′, 11.99″E] a settlement in Nigeria. The extraction process of fibre was done by beating the bark of the husk in the dry state and by soaking in water and chemical (NaOH). The percentage concentration of NaOH chemical used was 1%. The mixing of the fibre and concrete was done based on a percentage of the weight of the cement to be used in line with [12]. The fibre content varied within the range of 0.25–0.75.%. The slump test for the concrete samples was in line with [13]. The concrete panel of 100 mm × 100 mm × 40 mm dimensions were removed from the mould after a period of 24 h and were submerged into curing tank for 7, 14, 21 and 28 days in line with [14], [15]. A wooden box of (100 mm × 100 mm × 500 mm) required dimension was fabricated. The wooden box was then placed on a flat surface, sample of concrete panel was placed at the center of the wooden box. The loudspeaker and digital sound level meter are placed at extreme ends of the box. The digital sound meter is placed such that the sound that passes through is measured at the maximum level. The speaker specification is S200 model, power consumption: 5 V (volt) DC (Direct current) up to 1.0 A 4 Ω 3 W speaker. The frequency was generated by a frequency generator application from the Samsung play store.
